# Can we predict early 7-day readmissions using a standard 30-day hospital readmission risk prediction model?

**DOI:** 10.1186/s12911-020-01248-1

**Published:** 2020-09-15

**Authors:** Sameh N. Saleh, Anil N. Makam, Ethan A. Halm, Oanh Kieu Nguyen

**Affiliations:** 1 Department of Internal Medicine, University of Texas Southwestern Medical Center, Dallas, USA; 2 Department of Population and Data Sciences, University of Texas Southwestern Medical Center, Dallas, USA; 3 Division of Hospital Medicine, San Francisco General Hospital, University of California San Francisco, San Francisco, USA

**Keywords:** Hospital utilization, Healthcare quality improvement, Early readmissions, Hospital medicine, Care transitions, Predictive model, Clinical decision support

## Abstract

**Background:**

Despite focus on preventing 30-day readmissions, early readmissions (within 7 days of discharge) may be more preventable than later readmissions (8–30 days). We assessed how well a previously validated 30-day EHR-based readmission prediction model predicts 7-day readmissions and compared differences in strength of predictors.

**Methods:**

We conducted an observational study on adult hospitalizations from 6 diverse hospitals in North Texas using a 50–50 split-sample derivation and validation approach. We re-derived model coefficients for the same predictors as in the original 30-day model to optimize prediction of 7-day readmissions. We then compared the discrimination and calibration of the 7-day model to the 30-day model to assess model performance. To examine the changes in the point estimates between the two models, we evaluated the percent changes in coefficients.

**Results:**

Of 32,922 index hospitalizations among unique patients, 4.4% had a 7-day admission and 12.7% had a 30-day readmission. Our original 30-day model had modestly lower discrimination for predicting 7-day vs. any 30-day readmission (C-statistic of 0.66 vs. 0.69, *p* ≤ 0.001). Our re-derived 7-day model had similar discrimination (C-statistic of 0.66, *p* = 0.38), but improved calibration. For the re-derived 7-day model, discharge day factors were more predictive of early readmissions, while baseline characteristics were less predictive.

**Conclusion:**

A previously validated 30-day readmission model can also be used as a stopgap to predict 7-day readmissions as model performance did not substantially change. However, strength of predictors differed between the 7-day and 30-day model; characteristics at discharge were more predictive of 7-day readmissions, while baseline characteristics were less predictive. Improvements in predicting early 7-day readmissions will likely require new risk factors proximal to day of discharge.

## Background

Despite intense focus on preventing 30-day readmissions, early readmissions within the first 7 days of hospital discharge may be more preventable than later readmissions (8–30 days post-discharge) [1–7]. Early readmissions are more closely related to potential gaps in care during the index hospitalization [4] or reflect premature discharge [7]. Identifying patients at risk for early, rather than later readmissions may be a more effective strategy to tailor resource-intensive transitional care interventions to prevent readmissions. However, current risk prediction models often only identify patients at risk for 30-day readmission [8–10] and often fail to use electronic health record (EHR) data effectively to allow for real-time operationalization of the model [10, 11]. There is a paucity of research developing prediction models for adult 7-day readmissions [8, 12], which may be due to federal financial penalties tied to 30-day readmissions. Yet, to our knowledge, no study has investigated if a 30-day model can be reapplied effectively to predict the important subset of 7-day readmissions. Therefore, we assessed how well a validated 30-day EHR-based readmission risk prediction model [13] would predict early 7-day readmissions, and whether there were differences in the strength of predictors for 7-day versus 30-day readmissions.

## Methods

We conducted an observational cohort study of consecutive hospitalizations by adults ≥18 years from November 2009 to October 2010 using electronic health record (EHR) data from 6 diverse hospitals in north Texas, including safety-net, academic, and community sites. We included index hospitalizations of patients who were alive 30 days post-discharge. Patients who died in the hospital, were transferred to another hospital, or left against medical advice were excluded. The primary outcome was all-cause non-elective 7-day hospital readmissions within a 100-mile radius of Dallas, Texas (includes 75 acute care hospitals), which were retrieved from an all-payer regional hospitalization database. The previously validated 30-day readmission multivariate logistic regression model was developed using a 50–50 derivation-validation split. Multiple groups of candidate predictors including socioeconomic, admission day, hospital stay, and discharge day variables were included in development of the model. All variables were available in the EHR for all participating hospitals and were plausible based on previous literature and clinical expertise. Further details about model development have been previously published [13].

Using the same derivation cohort, we ran a multivariate logistic regression to re-derive model coefficients for the same predictors from our validated 30-day readmission model [13] (also developed from the same cohort) to optimize prediction of 7-day readmissions. Using the same cohort allows for direct comparison of model performance and changes in coefficient direction and magnitude between models. We used the validation cohort to compare the discrimination and calibration of our 7-day readmission model with our original 30-day model to predict 7-day readmissions. Discrimination was assessed using the C statistic, which measures the goodness of fit of a logistic regression model by determining the probability a patient who experienced an event (in this case, a readmission) had a higher model risk than a patient who had not experienced the event. Calibration was evaluated by comparing predicted to observed probabilities for quintiles of risk. We calculated the categorical net reclassification improvement (NRI), which is the absolute net gain in correctly reclassified predictions of high (top 2 risk quintiles) and low risk (bottom 3 quintiles) for the 7-day readmission model compared to the 30-day model [14]. To examine which factors were more (or less) weighted in the7-day readmission model, we evaluated the percent change in coefficients between the two models, using the 30-day model as reference. Odds ratios for variables were estimated from the coefficients of each logistic regression model.

## Results

**Fig. 1:**
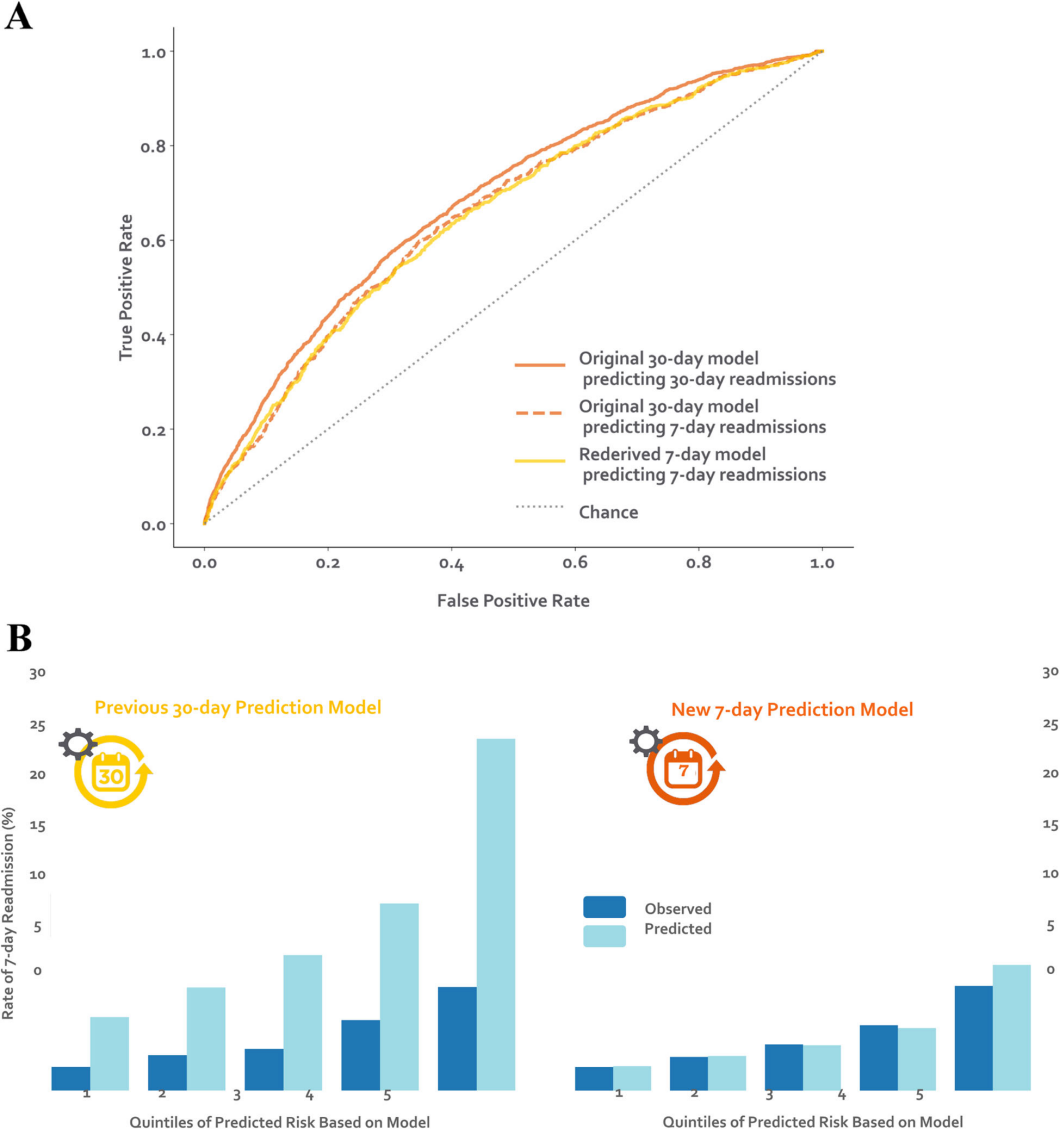
Model Performance of the 7-day versus 30-day Readmission Models. **a** Receiver operating characteristic (ROC) curves. The original 30-day model had modestly lower discrimination for predicting 7-day versus 30-day readmission (C-statistic of 0.66 vs. 0.69, *p* ≤ 0.001). Our re-derived 7-day readmission model had similar discrimination as the original 30-day model for predicting 7-day readmissions (C-statistic of 0.66 vs. 0.66, *p* = 0.38). **b** Calibration. To predict 7-day readmissions, the new 7-day prediction model had better calibration than the original 30-day prediction model across all quintiles of risk, but risk stratification was similar

Of 32,922 index hospitalizations among unique patients, 4.4% had a 7-day readmission and 12.7% had a 30-day readmission. Compared to those with 8-to-30-day readmissions, fewer patients with 7-day readmissions had one or more hospitalizations in the past year (43.3% vs. 47.7%, *p* = 0.01). On discharge, patients with 7-day readmissions had a higher proportion of one or greater vital sign instability (25.5% vs. 22.6%, *p* = 0.03) and sodium < 135 mEq/L (21.9% vs. 18.4%, *p* = 0.007) (Table 1). Our original 30-day model had modestly lower discrimination for predicting 7-day versus 30-day readmission (C-statistic of 0.66 vs. 0.69, *p* ≤ 0.001) (Fig. 1a). Our 7-day readmission model had similar discrimination as the original 30-day model for predicting 7-day readmissions (C-statistic of 0.66 vs. 0.66, *p* = 0.38) but improved calibration, particularly for the highest risk quintile (Fig. 1b). The 7-day model did not have better reclassification (NRI = 0.006, 95% CI: − 0.104 – 0.116).

**Table 1:** Descriptive characteristics of 8-to-30-day vs. 7-day readmissions

	8-to-30-day Readmissions *N* = 2747 (8.3%)	7-day Readmissions *N* = 1447 (4.4%)	*P*
**Baseline factors**			
Age in years^b^	65 (52–78)	66 (53–79)	0.04
Widow^a^	465 (16.9)	245 (16.9)	0.97
Medicaid^a^	313 (11.4)	151 (10.4)	0.37
≥ 1 ED visit in past year^a^	1024 (37.3)	540 (37.3)	0.99
≥ 1 hospitalization in past year^a^	1301 (47.4)	627 (43.3)	0.01
**Factors from admission day**			
Nonelective admission^a^	2447 (89.1)	1280 (88.4)	0.58
Charlson Comorbidity Index^b^	0 (0–3)	0 (0–3)	0.02
*Laboratory abnormalities within 24 h of admission*			
Albumin < 2 g/dL^a^	57 (2.1)	37 (2.6)	0.37
Albumin 2–3 g/dL^a^	590 (21.5)	292 (20.2)	0.35
Aspartate aminotransferase > 40 U/L^a^	469 (17.1)	259 (17.9)	0.53
Creatine phosphokinase < 60 mcg/L^a^	400 (14.6)	213 (14.7)	0.93
Mean corpuscular volume > 100 fL/red cell^a^	184 (6.7)	71 (4.9)	0.03
Platelets < 90 × 10^3^/μL^a^	150 (5.5)	68 (4.7)	0.33
Platelets > 350 × 10^3^/μL^a^	369 (13.4)	182 (12.6)	0.46
Prothrombin time > 35 s^a^	42 (1.5)	19 (1.3)	0.67
**Factors from hospital stay**			
Discharge to hospice^a^	17 (0.6)	14 (1.0)	0.29
*Hospital complications*			
*Clostridium difficile* infection^a^	20 (0.7)	13 (0.9)	0.68
Pressure ulcer^a^	54 (2.0)	29 (2.0)	0.97
Venous thromboembolism^a^	39 (1.4)	22 (1.5)	0.90
**Factors from discharge day**			
*Laboratory abnormalities at discharge*			
Blood urea nitrogen > 20 mg/dL^a^	1134 (41.3)	634 (43.8)	0.12
Sodium < 135 mEq/L^a^	505 (18.4)	317 (21.9)	0.007
Hematocrit <= 27%^a^	372 (13.5)	190 (13.1)	0.75
≥1 vital sign instability at discharge^a^	621 (22.6)	369 (25.5)	0.03
Length of stay^b^	5 (3–8)	5 (3–8)	0.37

*Abbreviation*: *ED* Emergency department

^a^ Denotes a binary variable, which is shown as number (%)

^b^ Denotes a numerical variable, which is shown as median (interquartile range)

**Fig. 2:**
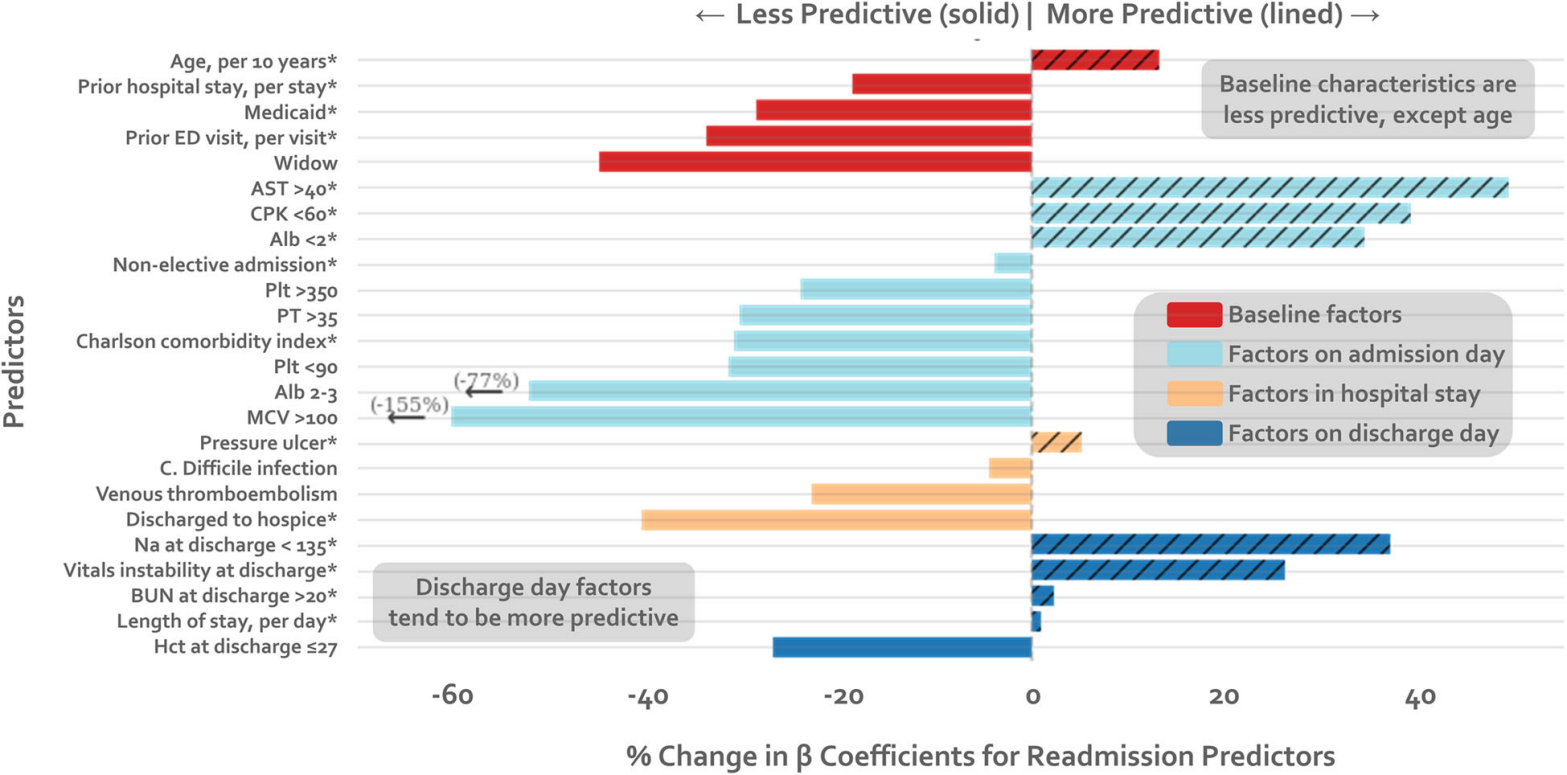
Change in Strength of Predictors. The percent change in β coefficients between the original 30-day model and the re-derived 7-day model is shown for each predictor included in the model. Predictors are grouped according to the timing of their availability, including baseline characteristics prior to the index hospitalization (red), factors on hospital admission (light blue), factors during hospital stay (gold) and factors on discharge day (dark blue). Values to the right of the vertical dashed line at 0, shown in striped color, indicate factors that are more predictive of early readmission. Values to the left of the dashed line showed in solid color, indicate factors that are less predictive. Variables that are still statistically significant in the 7-day model are denoted with an asterisk (*)

When comparing strength of predictors between the two models, clinical characteristics at discharge, such as sodium and vital sign instability, were more strongly predictive of 7-day readmissions compared to 30-day readmissions (Table 2). Baseline characteristics (Medicaid and prior utilization), were less predictive of 7-day readmissions. Factors on admission and during the hospital stay also tended to be less predictive. Mean corpuscular volume > 100 fL/red cell (− 155%) and Albumin 2-3 g/dL (− 77%) had the greatest negative difference in coefficients from the 30-day model to the 7-day model (Fig. 2). The strongest statistically significant positive predictor of readmission was *Clostridium difficile* infection in the 30-day model and Albumin < 2 g/dL in the 7-day model. The strongest negative predictor in both models was discharge to hospice care. While all variables were statistically significant in the 30-day model, only 15 of the 24 variables were in the 7-day model.

**Table 2:** Comparing strength of predictors of 30-day vs. 7-day readmissions^a^

	Adjusted Odds Ratio (95% CI)	
	Original 30-day Readmission Model	New 7-day Readmission Model
**Baseline factors**		
Age in years, per 10 years^b^	1.07 (1.04–1.10)	1.08 (1.03–1.14)
Widow	1.27 (1.11–1.45)	1.13 (0.92–1.40)
Medicaid^b^	1.55 (1.31–1.83)	1.37 (1.06–1.78)
Prior ED visit, per visit^b^	1.04 (1.02–1.06)	1.03 (1.01–1.04)
Prior hospitalization, per hospitalization^b^	1.16 (1.12–1.20)	1.13 (1.08–1.18)
**Factors from admission day**		
Nonelective admission^b^	1.42 (1.22–1.65)	1.40 (1.09–1.80)
Charlson Comorbidity Index, per point^b^	1.06 (1.04–1.09)	1.04 (1.01–1.08)
*Laboratory abnormalities within 24 h of admission*		
Albumin < 2 g/dL^b^	1.52 (1.05–2.21)	1.75 (1.06–2.87)
Albumin 2–3 g/dL	1.20 (1.06–1.36)	1.04 (0.86–1.27)
Aspartate aminotransferase > 40 U/L^b^	1.21 (1.06–1.38)	1.34 (1.09–1.63)
Creatine phosphokinase < 60 mcg/L^b^	1.28 (1.11–1.46)	1.40 (1.14–1.72)
Mean corpuscular volume > 100 fL/red cell	1.32 (1.07–1.62)	0.86 (0.60–1.23)
Platelets < 90 × 10^3^/μL	1.56 (1.23–1.97)	1.36 (0.94–1.96)
Platelets > 350 × 10^3^/μL	1.24 (1.08–1.44)	1.18 (0.94–1.49)
Prothrombin time > 35 s	1.92 (1.73–2.90)	1.57 (0.84–2.94)
**Factors from hospital stay**		
Discharge to hospice	0.23 (0.13–0.40)	0.41 (0.20–0.86)
*Hospital complications*		
Clostridium difficile infection^b^	2.03 (1.18–3.48)	1.96 (0.96–4.00)
Pressure ulcer^b^	1.64 (1.15–2.34)	1.68 (1.01–2.79)
Venous thromboembolism	1.55 (1.03–2.32)	1.40 (0.76–2.58)
**Factors from discharge day**		
*Laboratory abnormalities at discharge*		
Blood urea nitrogen > 20 mg/dL^b^	1.37 (1.24–1.52)	1.38 (1.17–1.62)
Sodium < 135 mEq/L^b^	1.34 (1.18–1.51)	1.49 (1.24–1.79)
Hematocrit <= 27%	1.22 (1.05–1.41)	1.16 (0.92–1.46)
Vital sign instability at discharge, per instability^b^	1.25 (1.15–1.36)	1.32 (1.17–1.50)
Length of stay, per day^b^	1.06 (1.04–1.07)	1.06 (1.04–1.08)

*Abbreviation*: *ED* Emergency department

^a^ Values reflect adjusted odds ratios and 95% CI for each variable after adjustment for all other variables listed in the table separately for our re-derived early 7-day model and our original validated 30-day readmission model

^b^ Indicates variables that are still statistically significant in the 7-day model. All variables in the 30-day model were statistically significant

## Discussion

While growing research [1–7] supports that 7-day readmissions are more preventable than 30-day readmissions, 30-day readmissions continue to dominate the readmission prediction space. Few studies explicitly have developed prediction models for 7-day readmissions [8, 12]. Herein, we provide empirical evidence that a previously validated, multi-condition 30-day EHR-based readmission risk prediction model can also be used to predict 7-day readmissions.

Performance for the original 30-day risk prediction model was not substantially different compared to a re-derived 7-day model. Reweighting coefficients for predictors led to slightly improved calibration, but risk stratification and reclassification of risk were similar. Therefore, until more robust 7-day specific readmission models are developed, this EHR-based 30-day model can be applied as an effective stopgap to also predict and target more preventable 7-day readmissions.

While overall model performance was similar, strength of predictors for 7-day versus 30-day readmission differed. Characteristics at discharge were more predictive of early 7-day readmissions, while baseline characteristics were less predictive. This is consistent with prior research suggesting that early readmissions are more likely to be related to clinical stability on discharge than 30-day readmissions [1–6]. Further research is needed to more conclusively compare predictive differences between those variables that were statistically significant in the 30-day model, but not in the 7-day model. Our study supports the inclusion of additional risk factors proximal to day of discharge such as the quality of transition of care planning (e.g. timely outpatient follow-up, medication reconciliation, and dispensing on discharge) to optimize performance of future 7-day readmission risk prediction models. Further optimizing risk prediction would enable hospitals to more efficiently target and reduce those readmissions that are potentially the most preventable.

Our study benefitted from the large, multicenter diverse cohort and high-quality ascertainment of readmissions beyond the index hospital. The use of rich, ubiquitous EHR data allows for real-time operationalization of the model wherein patients can be identified for intervention before they are discharged as is used in our hospital system [15]. Furthermore, since we used the original cohort from which the 30-day readmission model was developed [13], we were uniquely positioned to isolate the ability of a 30-day readmission model to predict early 7-day readmissions by avoiding any differences in model performance stemming from changes in the study population itself. Study limitations include uncertain generalizability to other settings and use of data before federal penalties for hospital readmission were in effect.

## Conclusions

A previously validated 30-day readmission model can be used as an effective stopgap for prediction of 7-day readmissions as model performance did not substantially change. However, strength of predictors differed between the 7-day and 30-day model informing future directions for predictive improvement, including greater focus on new risk factors proximal to day of discharge.

## Data Availability

The data that support the findings of this study are available from UT Southwestern Medical Center, but restrictions apply to the availability of these data, which were used under approval for the current study and so are not publicly available. Data are however available from the authors upon reasonable request and with permission of UT Southwestern Medical Center.

## Abbreviations

EHR: Electronic health record
NRI: Net reclassification improvement
ED: Emergency department
